# Reactive Extraction of Betaine from Sugarbeet Processing Byproducts

**DOI:** 10.1021/acsomega.2c07845

**Published:** 2023-03-13

**Authors:** Sinem Altinisik, Hani Zeidan, M. Deniz Yilmaz, Mustafa E. Marti

**Affiliations:** † Department of Chemical Engineering, Faculty of Engineering and Natural Sciences, Konya Technical University, 42075 Konya, Turkey; ‡ Department of Basic Sciences, Faculty of Engineering, Necmettin Erbakan University, 42140 Konya, Turkey

## Abstract

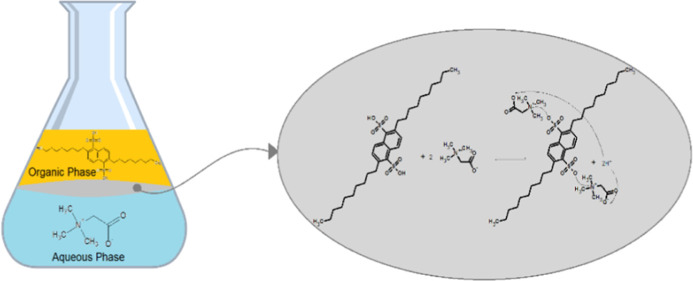

Betaine from natural sources is still preferred over its synthetic analogue in secondary industries. It is currently obtained by expensive separation means, which is one of the main reasons for its high cost. In this study, reactive extraction of betaine from sugarbeet industry byproducts, that is, molasses and vinasse, was investigated. Dinonylnaphthalenedisulfonic acid (DNNDSA) was used as the extraction agent, and the initial concentration of betaine in the aqueous solutions of byproducts was adjusted to 0.1 M. Although maximum efficiencies were obtained at unadjusted pH values (pH 6, 5, and 6 for aqueous betaine, molasses, and vinasse solutions, respectively), the effect of aqueous pH on betaine extraction was negligible in the range of 2–12. The possible reaction mechanisms between betaine and DNNDSA under acidic, neutral, and basic conditions were discussed. Increasing the extractant concentration significantly increased (especially in the range of 0.1–0.4 M) the yields, and temperature positively (but slightly) affected betaine extraction. The highest extraction efficiencies (∼71.5, 71, and 67.5% in a single step for aqueous betaine, vinasse, and molasses solutions, respectively) were obtained with toluene as an organic phase solvent, and it was followed by dimethyl phthalate, 1-octanol, or methyl isobutyl ketone, indicating that the efficiency increased with decreasing polarity. Recoveries from pure betaine solutions were higher (especially at higher pH values and [DNNDSA] < 0.5 M) than those from vinasse and molasses solutions, indicating the adverse influence of byproduct constituents; however, the lower yields were not due to sucrose. Stripping was affected by the type of organic phase solvent, and a significant amount (66–91% in single step) of betaine in the organic phase was transferred to the second aqueous phase using NaOH as the stripping agent. Reactive extraction has a great potential for use in betaine recovery due to its high efficiency, simplicity, low energy demand, and cost.

## 1 Introduction

Currently, betaine (also called trimethylglycine or glycine betaine) is produced by chemical synthesis and by isolation from sugarbeets or byproducts of beet processing. Betaine obtained by the latter process is preferentially termed natural betaine to distinguish it from its synthetic industrial analogue.^1^ Natural betaine has several advantages over synthetic betaine, and its use is preferred by the pharmaceutical, cosmetic, and healthcare industries.^2^ Moreover, betaine has a wide range of uses in the cleaning, agricultural, food (dietary supplements and beverages), and animal feed industries.^3−5^ Its global market value was about $3.3 billion in 2018 and is expected to surpass $5 billion by the end of 2027.

Betaine is a modified version of the simplest amino acid, glycine, and has similar zwitterionic properties.^6,7^ The ability of its methyl groups to serve as methyl donors in metabolism provides several advantages, for example, reduction of heart disease by remethylation of homocysteine.^8,9^ In addition, it functions as an osmoprotectant in plants, bacteria, and mammals, and it can prevent protein destabilization caused by high salt concentrations.^10,11^ Recently, rheological and zwitterionic properties of betaine have initiated new possibilities for its use in novel chemical syntheses and other functionalities.^12−14^

Bagasse, filter cake, and molasses are major byproducts of sugar production.^15^ Increased production costs have stimulated the evaluation of non-sugar streams from sugar purification processes as sources of value-added products,^16^ and betaine is one of these compounds.^6,17,18^ Molasses, the major and most valuable byproduct of beet sugar refining, has been the principal source of natural betaine.^19−21^ Vinasse, a byproduct of ethanol fermentation of molasses, is another important source of betaine.

Recovering betaine from natural sources is of great interest but also challenging. Most approaches have focused mainly on chromatography (e.g., ion exchange) and membrane technologies.^5,22^ Unfortunately, purchasing, operating, and maintaining these sophisticated processes are expensive. Moreover, dilute aqueous fractions cause more energy consumption in the evaporation stage. In addition, the high viscosities of sugar byproducts (high dilution ratio is required) and fouling due to their ingredients are major obstacles to recovering betaine by membrane separation.^23,24^ Moreover, stripping of the target product from membrane contactors is difficult and time-consuming. Recently, the cloud point extraction approach was applied for betaine recovery;^25^ however, use (and removal following the separation) of high amounts of salts and surfactants, high temperatures, and low yields are the main disadvantages of the method. Therefore, highly efficient, cost-effective, and, most importantly, scalable processes that can replace chromatographic separation methods are needed for commercial applications.

Reactive extraction has been identified as a promising technique for the recovery of biochemicals from dilute aqueous media^26^ and is preferred over other separation techniques due to its high efficiency, selectivity, operational practicality, and low-cost and energy demand.^27,28^ Moreover, in situ and continuous operational capability enable its use for commercial purposes. The technique has been evaluated for the recovery of metals, lanthanides, alcohols, antibiotics, phenols, vitamins, amines, and carboxylic acids from synthetically prepared aqueous solutions or complex media.^29−36^ The use of an extraction agent in the organic phase that can interact with the target product is the main difference between reactive extraction and its original technique. This couples the advantage of chemical extraction with physical extraction, resulting in higher recovery efficiencies.^27^ The solute targeted for transfer to the organic phase binds to the extractant at the interface to form a complex; for this reason, the process is also termed “complex extraction”.^37^ In the following step, the target product can be “stripped” or “back-extracted” using appropriate agents into another aqueous medium. The principal aqueous medium can be a fermentation broth, an industrial byproduct, a waste stream, or some other aqueous solutions containing the desired product. Selection of the organic phase components is critical since viscosity and hydrophobicity of the extraction agent, which significantly impact the extraction efficiency, are adjusted with the use of an organic phase diluent or mixture. In addition, optimization of the medium conditions is required.

There are few reports in the scientific literature that address the separation of betaine. Most of these used synthetically prepared aqueous solutions; however, sugar byproducts contain a wide variety of compounds that affect betaine recovery. The focus of this study was to investigate the recovery of betaine from sugarbeet byproducts, molasses and vinasse, by reactive extraction. Several alternatives were previously tested for use as the extractant in the extraction process, and screening trials identified dinonylnaphthalene disulfonic acid (DNNDSA) as the optimum extraction agent. This has been used to recover metals, for example, Ni^38^ and Cu,^39^ though, but not other chemicals. In extraction trials, DNNDSA was dissolved in four different organic solvents to form organic phases. In addition to the molasses and vinasse, trials were performed using aqueous pure betaine solutions, and the results were compared to assess the effect of betaine source and ingredients on the recovery. The objective was to determine with which components and under which conditions, the highest efficiency would be achieved. Moreover, the effects of various process parameters such as the concentration, temperature, and pH on the extraction efficiency were investigated. In the following step, the focus was to probe the recovery of betaine from an organic medium to the second aqueous phase containing a stripping agent.

## 2 Experimental Section

### 2.1 Materials

Anhydrous betaine (>98% pure, p*K*_b_ = 1.81) was supplied by Acros Organics, while DNNDSA (C_28_H_44_O_6_S_2_, 55 wt % in isobutanol) and sucrose were purchased from Sigma-Aldrich. 1-Octanol (Merck Co.), methyl isobutyl ketone (MIBK; Acros Organics), dimethyl phthalate (DMP, Acros Organics), and toluene (Merck Co.) were tested as organic phase diluent. The chemical structures of the components used in this study are given in Figure S1. Molasses and vinasse byproducts of sugarbeet industry were provided by the Konya Sugar Factory. Initial concentrations of betaine in molasses and vinasse were 7% (∼0.25 M) and 11% (∼0.37 M), respectively. Aqueous pure betaine-, molasses-, and vinasse solutions were prepared using ultra-high purity (UHP) water obtained from a Millipore Direct-Q 3V System. Initial pH values of the aqueous solutions were adjusted by using NaOH (Merck) or HCl (Merck). All chemicals were of analytical or higher grade and used without any treatment or further purification.

### 2.2 Equilibration Time

Prior to equilibrium studies, equilibration time was examined. Both organic [0.3 M DNNDSA in toluene (as an example)] and aqueous solutions (0.1 M betaine in water, molasses, and vinasse solutions) were shaken at 150 rpm and 298 K for 720 min. The concentration of betaine was screened at different contact times via multiple trials started simultaneously and terminated at varied periods.

### 2.3 Reactive Extraction (Equilibrium)

Liquid–liquid equilibrium experiments were carried out to determine extraction efficiency and equilibrium distribution of betaine between the organic and aqueous phases. DNNDSA was used as supplied (55 wt % or 0.997–1.0 M in isobutanol) and due to its high viscosity, further dilution was required. The initial concentration of DNNDSA in the organic solvents ranged from 0.1 to 0.5 M. Before the extraction trials, the betaine concentration in the byproduct solutions was adjusted to 0.1 M by diluting the molasses and vinasse 2.5 and 3.7 fold, respectively. The influences of initial pH and temperature were tested over the ranges of 2.0–12.0 and 298–338 K, respectively. The effect of carbohydrates in molasses on betaine recovery was investigated by adding sucrose to the aqueous media and its initial concentration was held constant and equal to that of betaine. Initial concentration of betaine in aqueous phases were equal to 0.1 M throughout the study. In pH trials, DNNDSA concentration in the organic phase was kept at 0.3 M, while it ranged from 0.1 to 0.5 M in the trials, where the effects of temperature and presence of sucrose were investigated. Equal volumes (5 mL each) of organic and aqueous phases were mixed and equilibrated by shaking at 150 rpm and 298 K for 1 h for aqueous betaine solutions and 5 h for molasses and vinasse solutions. At equilibrium, the mixture was allowed to settle for 15 min before and after centrifugation at 4000*g* for 5 min to obtain a clear phase separation. Aqueous phases were carefully recovered and analyzed for betaine concentration by high-performance liquid chromatography (HPLC).

### 2.4 Back Extraction (Stripping)

Back extraction trials for betaine recovery were performed using organic phases containing “betaine–DNNDSA” complexes, obtained from (forward) reactive extraction trials and aqueous solutions of 1.0 M NaOH, as the back extraction agent. Equal volumes (5 mL) of organic and second aqueous phases were mixed in an Erlenmeyer flask at 150 rpm and 298 K for 5 h, followed by centrifugation. The aqueous phase was recovered and analyzed for betaine by HPLC. Due to the difficulties in the analysis of the stripping phase, back extraction could be studied at only one concentration level.

### 2.5 Analysis

The concentration of betaine in aqueous-based solutions or phases before and after extraction trials was determined by HPLC (Agilent LC 1220) equipped with a UV detector and an Inertsil ODS-3V column (4.6 × 250 mm, GL Sciences). The mobile phase was 0.1% (v/v) H_3_PO_4_ at a flow rate of 1.0 mL/min. Analyses were done at ambient temperature and betaine was detected at 214 nm. In trials testing the effect of sugar, levels of betaine and sucrose determined by HPLC using a Kromasil NH_2_ column (4.6 × 250 mm, HiChrom). Analyses were carried out at room temperature and the mobile phase was 75% (v/v) aqueous acetonitrile at a flow rate of 1.0 mL/min. Sucrose and betaine were detected by refractive index (Agilent 1260 Infinity II) and UV, respectively.^4^

Betaine concentration in the organic phase was calculated by a mass balance around the aqueous phase. Equilibrium trials and chemical analyses were performed in replicates of 2 to 3. The averages were used to determine extraction efficiency (*E* %) and distribution coefficient (*K*_D_) using eqs 1 and 2. Similarly, betaine in the second aqueous phase after back extraction was determined by HPLC using an Inertsil ODS-3V column and UV detection. Back extraction efficiency (BE %) was calculated using eq 3.
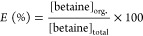
1
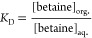
2
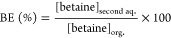
3

## 3 Results and Discussion

### 3.1 Equilibration Time

**Figure 1:**
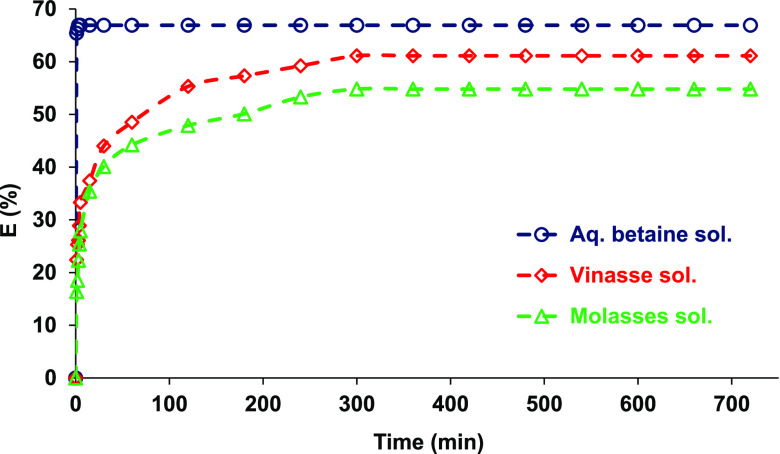
Equilibration time for the reactive extraction of betaine from several aqueous-based solutions using DNNDSA as the extraction agent ([betaine]_o_ = 0.1 M; [DNNDSA]_o_ = 0.3 M; solvent: toluene).

Before testing the effects of process variables, kinetic trials were performed to determine the time required to achieve equilibrium for the transfer of betaine from aqueous to organic phases. Trials were performed with three different aqueous phases: (a) aqueous (pure) betaine, (b) molasses, and (c) vinasse solutions at their unadjusted or natural pH values. Figure 1 shows that transfer of betaine from aqueous solution to organic phase occurs within minutes after the contact of the phases. However, the process was slower in vinasse and molasses solutions, requiring ∼300 min to reach equilibrium. Consistent results were observed for toluene, MIBK, 1-octanol, and DMP (data not shown). For this reason, the phases were mixed for 1- and 5 h for aqueous betaine solutions and byproduct solutions, respectively.

### 3.2 Effect of Solvent Type and DNNDSA Concentration

Molasses and vinasse are highly viscous byproducts and, without dilution, they are not suitable for use in reactive extraction. Dilution of sugarbeet processing byproducts for the recovery of betaine or other components is also required for other techniques such as chromatography, ion exchange, filtration, and other extraction types.^4,22,25^ Thus, the byproducts were diluted with UHP water resulting in initial betaine concentrations of 0.1 M. This was the highest (and equal) betaine concentration that can be reached by the dilution of the byproducts, which were obtained from a local sugar company. Initial amount of betaine in the byproduct solutions was kept constant for the comparisons of the yields. To observe and compare the effects of the byproduct sources on the reactive extraction yield, pure aqueous (0.1 M) betaine solutions were prepared and tested.

The contact of organic and aqueous phases during the extraction process is important for efficient extractive separations.^28,40^ Extraction agents can be viscous chemicals that they may not provide sufficient contact of the phases, resulting in lower extraction efficiencies. In addition, the extraction agent itself may not form a stable complex with the target solute. Since extraction of betaine occurs via the interfacial reaction between the extractant and target component, the extractants are dissolved in solvents (or diluents) to enhance the contact of the phases and adjust the properties of the organic phase for extraction. Organic solvents are preferred for the purpose due to their relatively low viscosities, water-immiscibilities, non-polarities, and hydrophobic nature, similar to the extraction agents that are usually used in the process.

DNNDSA used in this study was supplied at 55% wt in isobutanol; and its high viscosity prevents its use as it is received. Thus, it was dissolved in organic solvents, specifically 1-octanol, MIBK, toluene, and DMP to reduce its viscosity and corrosiveness prior to use. 1-Octanol is a known state-of-the-art solvent for reactive extraction of carboxylic acids, aminoacids, and metals in batch systems.^28,34,41^ MIBK is also used in extractive separation due to its functionality and relatively high polarity.^42,43^ Toluene and DMP were selected as non-polar solvents since they are widely used to recover non-polar solutes from natural and various sources.^43−46^ Hexane and heptane could not be used due to their inability to dissolve DNNDSA.

Preliminary trials showed that these four organic solvents alone, without DNNDSA, failed to physically extract betaine from aqueous solutions. Hence, they were individually used as organic phase diluents to recover betaine, and the initial DNNDSA concentration ranged from 0.1 to 0.5 M in the solvents. The efficiencies obtained with the four solvents were compared to determine the most effective and appropriate organic phase diluent. Organic and aqueous phases were mixed for sufficient time to reach extraction equilibrium.

**Figure 2:**
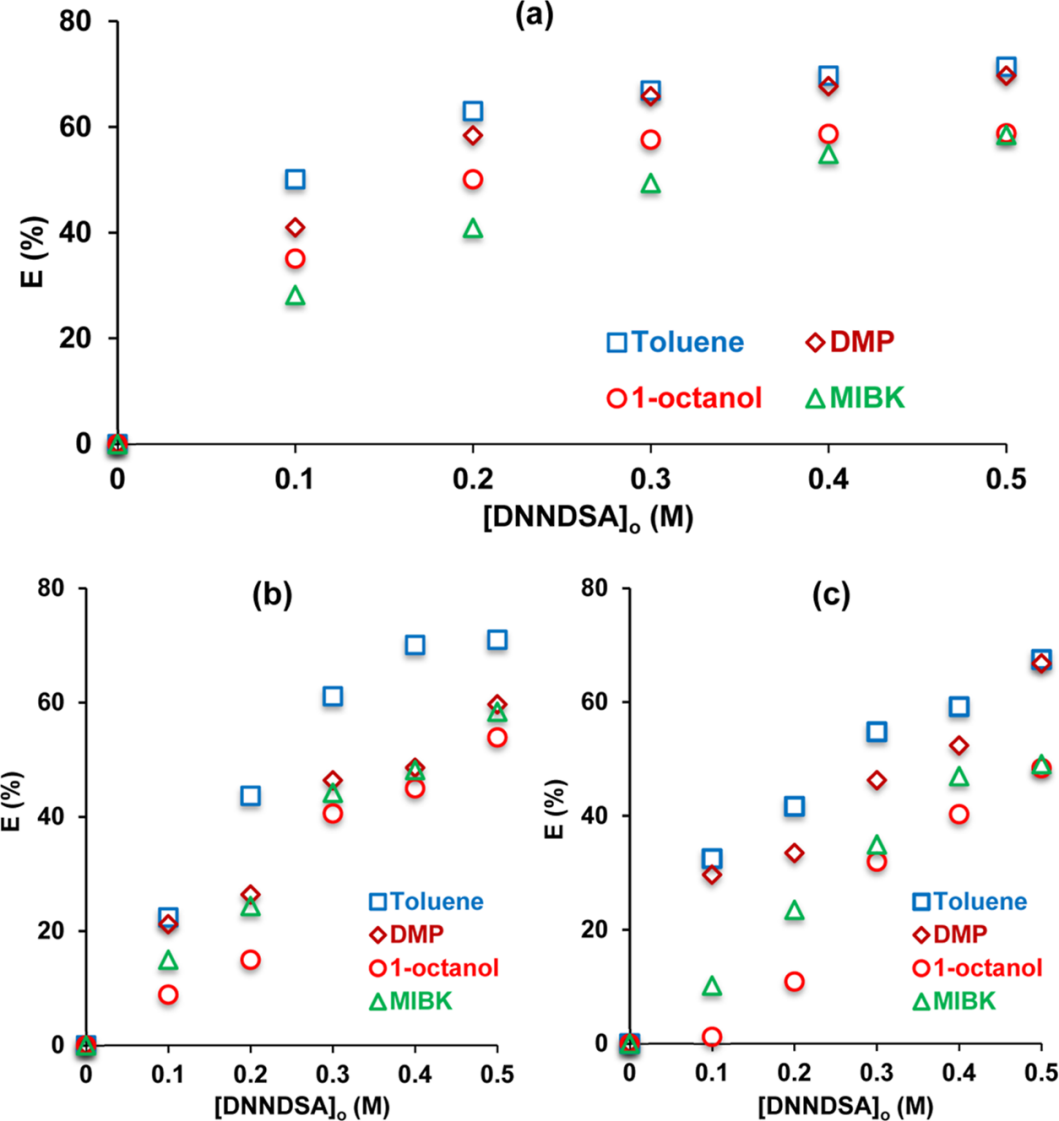
Effects of solvent type and DNNDSA concentration on the recovery of betaine from (a) aqueous betaine solution, (b) vinasse solution, and (c) molasses solution ([betaine]_o_ = 0.1 M).

Figure 2 shows the effects of solvent type and DNNDSA concentration on reactive extraction of betaine from aqueous betaine, vinasse- and molasses solutions. For all media studied, separation efficiency significantly increased at higher DNNDSA concentrations and the trend is consistent with all organic solvents tested (Table S1). The highest recovery yields were obtained with toluene (∼70%, *K*_D_ = 2.30) in the concentration range of 0.1–0.4 M for all aqueous solutions tested, followed by DMP, 1-octanol, and MIBK. The efficiencies with the latter three solvents were ∼1.5–20% lower than those obtained with toluene depending on the DNNDSA concentration and aqueous solution type.

Increasing DNNDSA concentration from 0.4 to 0.5 M did not significantly affect the betaine extraction from pure betaine solution; however, enhanced the recovery from byproduct solutions. At 0.5 M DNNDSA, the efficiencies converged for toluene and DMP as well as for 1-octanol and MIBK. This is likely due to poor contact of the phases because of relatively higher viscosity of the organic phases containing more DNNDSA. The trend was observed with acceptable exceptions for all aqueous based media and solvents used in the organic phases. The yields at 0.5 M in single step were ∼70–71.5% for toluene/DMP and ∼59% for 1-octanol/MIBK in aqueous (pure) betaine-; 71% for toluene and ∼54–60% for DMP/1-octanol/MIBK in vinasse-; and ∼67.5% for toluene/DMP and ∼49% for 1-octanol/MIBK in molasses solutions. These are higher than the yields obtained at the 0.1 M betaine concentration level using other separation techniques. The process was repeated twice to reach 68% in the cloud point extraction technique.^25^

In addition, more dilute (<0.1 M) betaine solutions are often required for the application of appropriate techniques. Literature reports indicate that higher yields are obtained at lower initial solute concentrations in reactive extraction systems.^28,32,35^ Escudero and Ruiz also mentioned that distribution coefficient reduced with the increase in initial betaine concentration.^4^ It is well known for extractions involving partitioning and complex formation, the more dilute the solute and the more extractant used, complex formation occurs to a greater extent; hence, the lower the betaine concentration, the higher the extraction yield.^47,48^ Therefore, efficiency may be higher with reactive extraction if initial betaine concentration is < 0.1 M. However, a high efficiency obtained at a relatively high initial solute concentration is an important advantage for the reduction of evaporation costs and commercialization of a separation technique.

The dielectric constant of a substance changes proportionally with its polarity^49^ and Table 1 lists the dielectric constants of the solvents tested in this study. Figure 2 shows that highest recovery efficiencies were obtained with the least polar solvent and yields decreased in the order of toluene > DMP > 1-octanol > MIBK, consistent with the order of their increasing dielectric constants. The data indicate that solvent polarity negatively affects the recovery process and the formation or solubility of the “betaine–DNNDSA” complex in the organic phase. It can be seen that DNNDSA is a non-polar compound (Figure S1). The complex made by DNNDSA and betaine is expected to be relatively polar than the extractant itself. However, its interaction with relatively polar organic solvents is still limited; and inactive solvents, for example, toluene and DMP, provide somehow a more favorable medium for the ion pair formation of sulfonic acid–quaternary ammonium (DNNDSA–betaine) complex and a higher distribution of betaine is achieved in the organic phase.

**Table 1:** Dielectric Constants of the Solvents Used in This Study and Maximum Extraction Efficiencies Obtained in Aqueous (Pure) Betaine, Molasses, and Vinasse Solutions

		maximum extraction efficiency (%)		
solvent	dielectric constant (ε)	aq. betaine sol.	molasses sol.	vinasse sol.
toluene	0.43	71.4	67.5	71.0
DMP	8.50	69.7	66.8	59.7
1-octanol	9.86	58.8	48.4	53.9
MIBK	13.10	58.5	49.1	58.4

### 3.3 Effect of Solution pH and Mechanism

**Figure 3:**
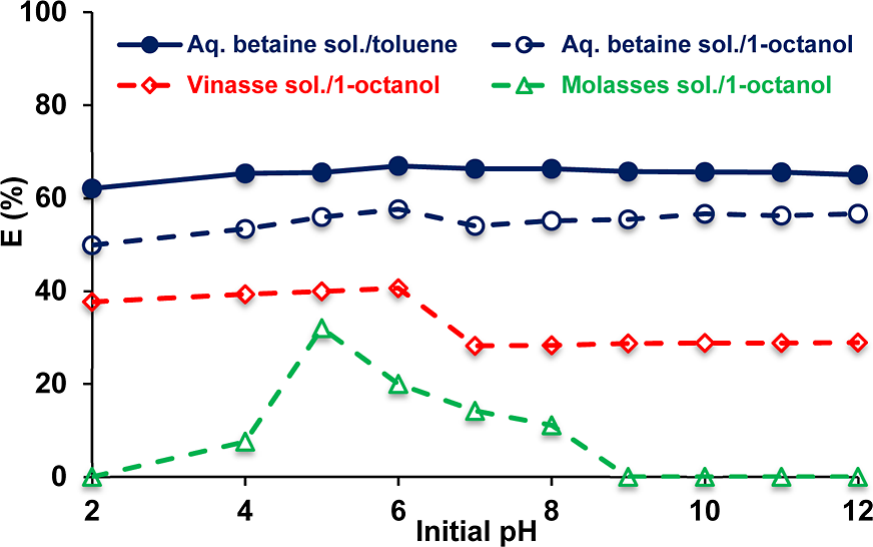
Effect of initial pH on the reactive extraction efficiency of betaine from aqueous-based solutions ([DNNDSA]_o_ = 0.3 M; [betaine]_o_ = 0.1 M; solvent: 1-octanol or toluene).

The effect of initial pH on the extraction efficiency was tested in the range of pH 2–12. The initial concentration of betaine was 0.1 M and unadjusted pH values of the aqueous betaine, molasses, and vinasse solutions were measured to be 6, 5, and 6, respectively. DNNDSA solutions (0.3 M) containing toluene or 1-octanol as organic phases were used in the trials. Figure 3 shows that extraction efficiencies were highest at the original or natural pH values of all betaine solutions tested. Moreover, yields were nearly identical or showed slight changes at all pH values of aqueous betaine solution (Table S1). Escudero and Ruiz mentioned that betaine recovery using a membrane contractor, where extraction and stripping steps are combined, should be operated at a pH lower than betaine dissociation constant (p*K*_b_), which is 1.81.^4^ However, the data showed that it is not required and the process can be operated without adjusting the pH in the range of 2–12 if these steps are carried out separately or not combined in one system. This is also consistent with the trend observed with the cloud point extraction method, where lower efficiencies were obtained with reducing or increasing the pH of the aqueous medium.^25^

For byproduct solutions, increasing initial pH reduced betaine extraction efficiency. The efficiency reduction was greater for molasses at higher pH values, suggesting that the extractant is hindered by or interacting with molasses constituents (having similar structure to betaine), which might be consumed or partially consumed during the fermentation of molasses. The maximum yields obtained in byproduct solutions were similar; however, efficiencies were higher for vinasse solution at all pH levels studied and no extraction was observed at pH ≥ 9 for molasses solution. Furthermore, yields with toluene were consistently higher than those with 1-octanol at all pH values tested for the pure betaine solution.

**Figure 4:**
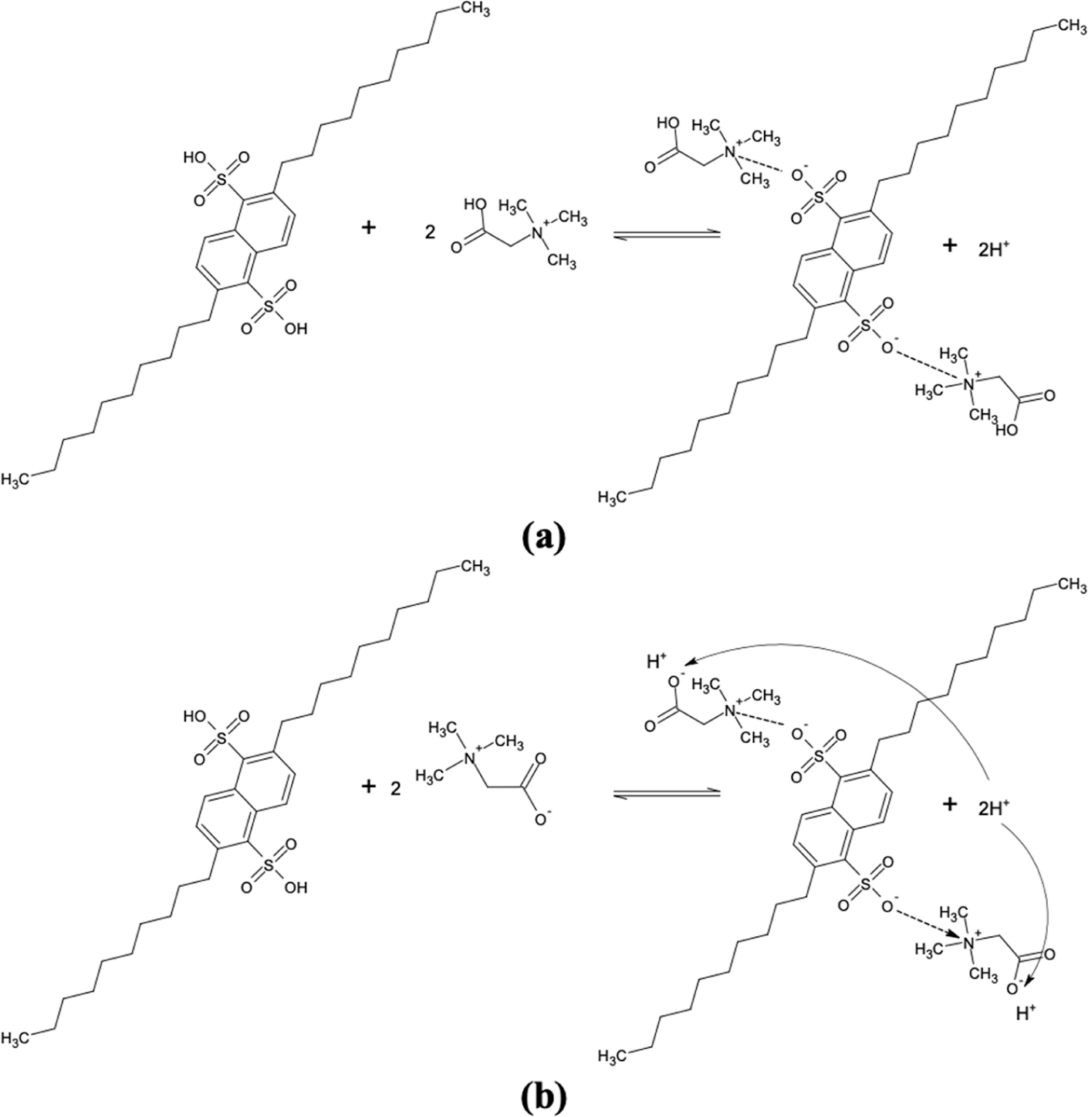
Reaction mechanism between betaine and DNNDSA (a) acidic system, pH between 2 and 5, and (b) neutral or basic system, pH between 6 and 12.

In view of the foregoing results, we propose a mechanism to explain the extraction process (Figure 4). Betaine can be cationic, zwitter-ionic or anionic in nature depending on the pH of the solution (Figure S2).^50−52^ However, the extraction efficiencies were almost identical at all pH values when aqueous betaine solutions were tested, indicating that binding may occur between the positively charged ammonium moiety of betaine and the negatively charged sulfonate moiety of DNNDSA according to the mechanism illustrated in Figure 4. The results outlined in Figure 3 are consistent with these mechanisms.^39,52^

The pH of the aqueous phase decreased during the reactive extraction of betaine from aqueous to organic phase, and the final pH reduced with the increase in DNNDSA concentration. The trend was observed for all organic phase diluents and aqueous-based solutions tested. However, final pH values were relatively higher for byproduct solutions and ranged between 2.9 and 4.8, while they reduced to 1.3–2.2 in pure betaine solutions. The difference in the final pH values is most likely due to the other components in the byproduct solutions.

### 3.4 Effect of Byproduct Ingredients and Sucrose

Molasses is a thick, syrupy sugar-rich residue that remains after purification of sucrose from sugarbeets and contains numerous components, for example, amino acids, minerals, organic acids, and so forth. It contains approximately 50% sucrose by weight and can be fermented by yeast to produce ethanol and the residue called vinasse with little-to-no sugar. Hence, vinasse also contains various chemical species. Effects of the components of molasses and vinasse on the recovery was evaluated by comparing the efficiencies obtained with aqueous betaine solutions and byproduct solutions.

**Figure 5:**
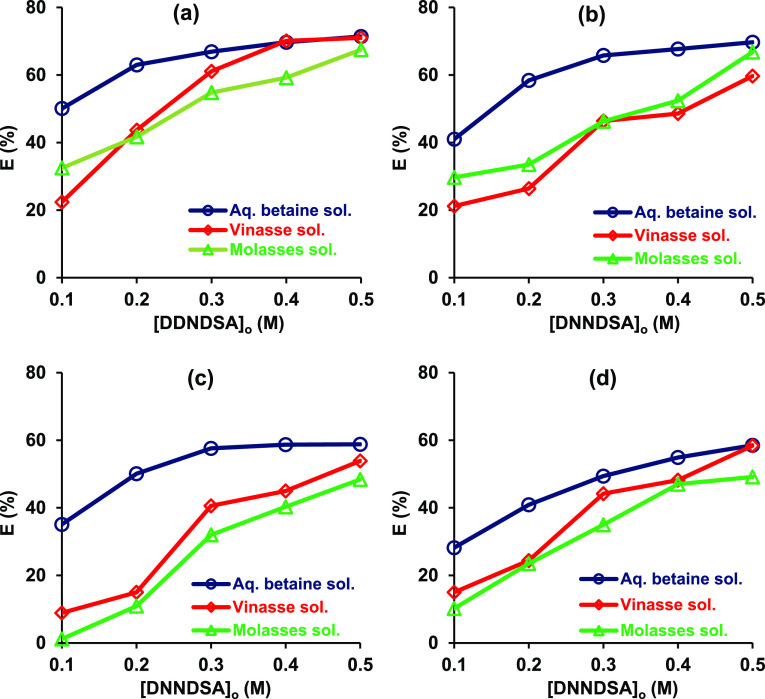
Effect of aqueous solution type on the recovery of betaine using DNNDSA dissolved in organic solvents ([betaine]_o_ = 0.1 M, Temp: 298 K, pH was unadjusted). (a) Toluene, (b) DMP, (c) 1-octanol, and (d) MIBK.

Figure 5 shows that recoveries obtained from aqueous betaine solutions were higher than those from molasses and vinasse solutions, indicating the adverse influence of byproduct constituents. Yields obtained from these two natural sources were comparable. An exception was observed at 0.4–0.5 M DNNDSA + toluene wherein efficiencies with vinasse solution were nearly equal to those in aqueous betaine solution. Efficiencies increased with DNNDSA concentration; however, those obtained in aqueous betaine solutions were very similar at 0.4- and 0.5 M DNNDSA. Thus, the differences between the yields in aqueous pure betaine and byproduct solutions reduced at higher DNNDSA concentrations. Efficiencies at 0.5 M were similar for all solution types and organic solvents tested.

**Figure 6:**
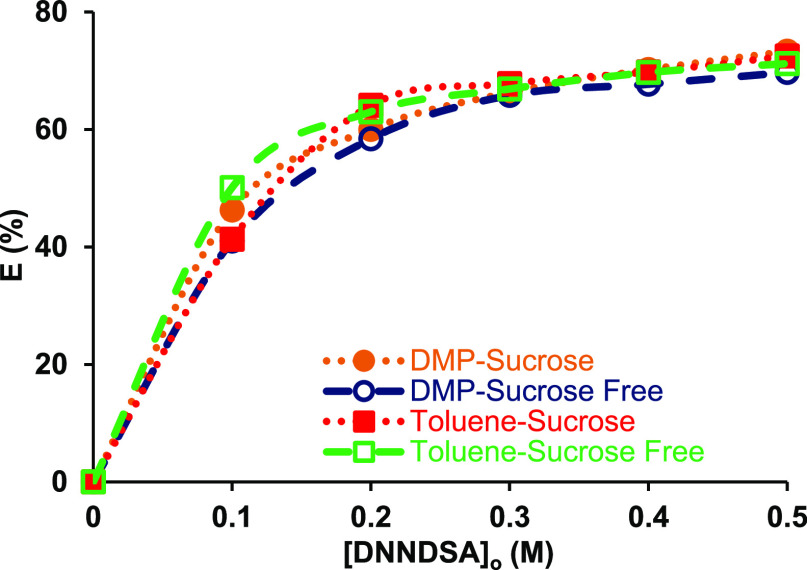
Effect of the presence of sucrose in aqueous media on the reactive extraction of betaine from pure aqueous betaine solution using DNNDSA dissolved in toluene or DMP ([betaine]_o_ = 0.1 M, Temp: 298 K, pH: 6 (unadjusted)).

The effect of sucrose on extraction of aqueous betaine was examined. Figure 5 shows that the highest betaine recoveries obtained from aqueous sucrose-free solutions were 71.4 and 69.7% with toluene and DMP, respectively, whereas, yields from sucrose-containing solutions were 72.5 and 73.3%, respectively. Clearly, for both toluene and DMP at all DNNDSA concentrations studied, sucrose in the aqueous phase has an insignificant effect on betaine extraction, which is consistent with the literature.^4^ This is also consistent with the trend observed in Figure 6 as the efficiencies with molasses and vinasse solutions were very similar. Although their compositions differ, substances they contain in common may be responsible for yield reductions.

### 3.5 Effect of Temperature

**Figure 7:**
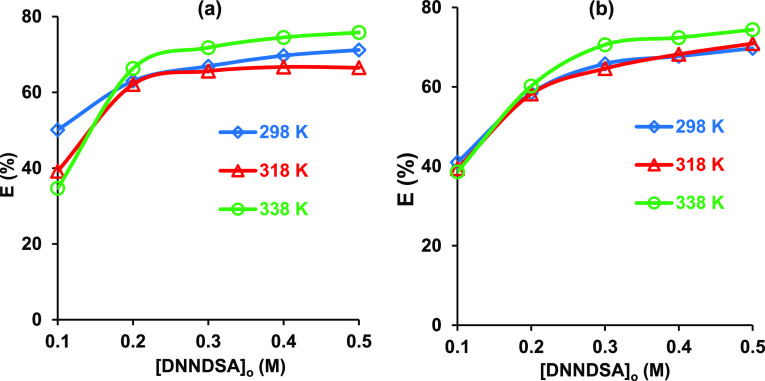
Effect of temperature on the reactive extraction of betaine from aqueous-based solution. Solvent: (a) DMP and (b) toluene ([betaine]_o_ = 0.1 M, pH was unadjusted).

To investigate the effect of temperature on the reactive extraction of betaine, the experiments were conducted at different temperatures by using two different organic phases, namely, toluene (Figure 7a) and DMP (Figure 7b). Figure 7 shows that the efficiency increased with the increase in temperature; however, it is consistent with the literature that the effect was negligible through the range tested.^4,27,28^ Several possibilities may account for the upward trend; (i) the extraction process may be endothermic in nature, (ii) diffusion resistance in the system might decrease with higher temperatures, (iii) a higher heat-induced reaction rate causes a shift to the formation of the DNNDSA–betaine complex in the organic phase, and (iv) the viscosities of the individual components in these organic phases decrease with temperature. Yu and Liu reported a similar trend for the use of DNNSA to extract magnesium from phosphoric acid and stated that the viscosity of the organic phase significantly reduced with the temperature.^53^ Although the highest efficiencies were achieved at 338 K, the additional energy required to raise the temperature does not provide an attractive efficiency advantage since the positive effect is insignificant.

### 3.6 Back Extraction

In the first step of the recovery process, betaine was successfully transferred from the aqueous-based solutions to the organic phase using DNNDSA dissolved in an organic solvent. Recoveries using non-polar solvents, toluene and DMP, were higher than those with polar solvents (1-octanol and MIBK). However, betaine that is complexed with DNNDSA must be recovered from the extraction agent and organic solution. Distillation is frequently used in industry for similar purposes; however, recent increases in energy prices could significantly increase the cost and price of betaine. Therefore, using a low-cost and efficient stripping agent for betaine recovery from the organic phase is an attractive option. This would increase the availability of betaine for use in industrial applications; moreover, a betaine-free organic phase can be re-used in consecutive extraction processes, thereby lowering recovery costs. Due to the difficulties in the analyses of the back-extracted aqueous phases, only NaOH (at a concentration level of 1.0 M) was tested as the stripping agent and trials were performed using only aqueous betaine solutions.

Back extraction trials were performed using the organic phases obtained from reactive extraction experiments where initial concentrations of DNNDSA and betaine were 0.5 and 0.1 M, respectively. Effect of the organic phase diluent on the back extraction or stripping efficiency was investigated. Thus, organic phases including toluene, DMP, and 1-octanol as diluents were used in back extraction trials. Reactive extraction efficiencies obtained with 0.5 M DNNDSA in these diluents were 71.4-, 69.7-, and 58.8%, respectively (Figures 2 and 5).

**Figure 8:**
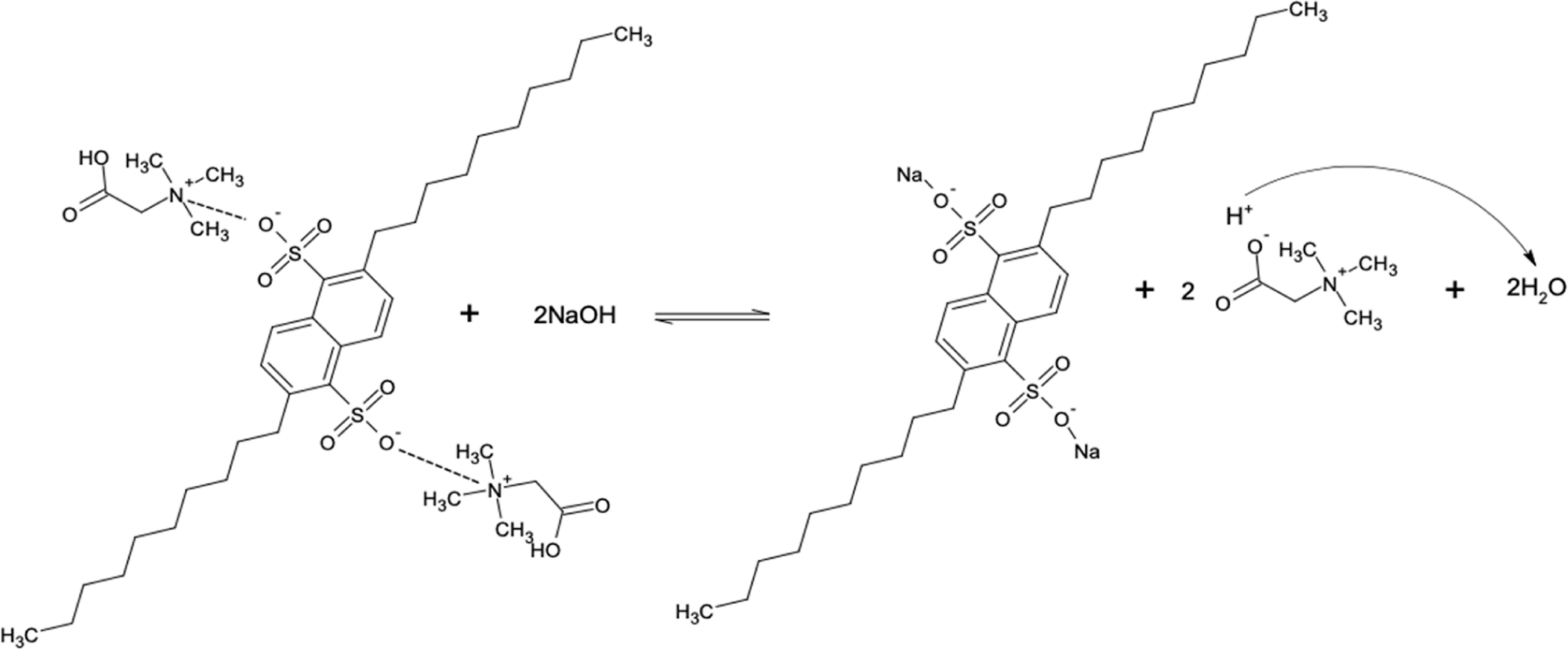
Proposed mechanism for back extraction of betaine using NaOH ([back extractant, NaOH] = 1 M, [betaine]_o_ = 0.1 M).

Table 2 shows that stripping was affected by type of the organic phase solvent and highest efficiency was obtained when 1-octanol was used in the process, and it was followed by DMP and toluene and the yields were 66.4-, 71.9-, and 91.4% for these solvents, respectively. Consistently, stripping efficiency reduced with the decrease in polarity. Considering the number of moles transferred to the second aqueous phase, efficiency with 1-octanol was 6 and 13.6% were higher than that with DMP and toluene, respectively (since reactive extraction yields were different). Decrease in the pH of the (second) aqueous phase during the stripping process was negligible (from 14 to ∼13.1–13.5) due to the relatively high concentration of NaOH. Relatively stronger interaction between the “betaine–DNNDSA complex” and the “non-polar solvent”, toluene or DMP, might explain the lower stripping efficiencies. Moreover, lower betaine concentration in 1-octanol due to the lower reactive extraction yields might be responsible for higher stripping efficiency. The possible stripping mechanism is illustrated in Figure 8. Following this step, highly purified betaine can be obtained by removing water from the aqueous solution using an appropriate technique such as crystallization and/or evaporation.

**Table 2:** Effect of Organic Phase Diluent on the Stripping or Back Extraction of Betaine from Organic Media to the Second Aqueous Phase Using 1.0 M NaOH

organic phase solvent	back extraction efficiency (%)
1-octanol	91.4
DMP	71.9
toluene	66.4

The data present that 47.4–53.7% of the betaine initially presents in the aqueous phase could be recovered in only one “reactive extraction + stripping” step. Escudero and Ruiz mentioned that 46% of the betaine in the sucrose-free system could be recovered by membrane contactor. Since the highest recovery yield was only about 42% with cloud point extraction, the process was repeated three times before recovering betaine from the extract phase; therefore, single stage efficiency was not reported. However, the amount of betaine recovered by reactive extraction can also be increased by repeating the process or using a multi-stage system.

## 4 Conclusions

The recovery of the betaine from aqueous betaine, vinasse, and molasses solutions by reactive extraction was studied in this study. The extractant, DNNDSA, was dissolved in four different organic solvents (toluene, DMP, 1-octanol, and MIBK) to form organic phases wherein its concentration ranged from 0.1 to 0.5 M. The molasses and vinasse were diluted 2.5 and 3.7 fold, respectively, to adjust the betaine concentration to 0.1 M, as in the pure betaine solution. Reactive extraction systems using byproduct solutions required ∼5 h to achieve equilibrium, while the one including aqueous pure betaine solution reached it within minutes after the contact of the phases. Increasing solution pH reduced the extraction efficiency in molasses and vinasse solutions but not in pure betaine solution. Highest yields were obtained at the unadjusted (natural) pH values of these solutions. Efficiencies significantly increased at higher DNNDSA concentrations. Highest recoveries were obtained with toluene, a non-polar (inactive) solvent, and its superiority was demonstrated in the concentration range of 0.1–0.4 M DNNDSA. The yields at 0.5 M were ∼70–71.5% for toluene/DMP and ∼59% for 1-octanol/MIBK in aqueous betaine-; 71% for toluene and ∼54–60% for DMP/1-octanol/MIBK in vinasse-; and ∼67.5% for toluene/DMP and ∼49% for 1-octanol/MIBK in molasses solutions. The lower efficiencies obtained in byproduct solutions (compared to that in pure betaine solution) was not due to the presence of sucrose in the media, also indicating the selectivity of DNNDSA for betaine extraction in a sugar-containing system.

The results indicate that 47.4–53.7% of the betaine initially present in the aqueous phase could be recovered in only one “reactive extraction + stripping” step; more betaine can be recovered using a multi-stage separation system. This study shows that reactive extraction can be used to recover betaine from sugarbeet industry byproducts using an appropriate extraction agent dissolved in a non-polar solvent or media.

## Supporting Information Available

Significance of process parameters on the extraction efficiency; structures of the chemicals used; and behavior of betaine in acidic, neutral, and basic solutions (PDF)

## Abbreviations

BE %: back extraction efficiency
BH^+^: betaine cationic form
DMP: dimethyl phthalate
DNNDSA: dinonylnaphthalene disulfonic acid
*E* %: extraction efficiency
*K*_D_: distribution coefficient
*K*_a_: dissociation constant of DNNDSA (m^3^/kmol)
MIBK: methylisobutyl ketone
*V*: volume of the phase, mL
[ ]: concentration (M)
ε: dielectric constant

## Subscripts

aq.: aqueous phase
o: at initial (for concentration)
org.: organic phase
sol.: solution
