# Supplementary material for: Reactive Extraction of Betaine from Sugarbeet Processing Byproducts
Source: ACS Omega. 2023 Mar 13;8(12):11029–38. doi: 10.1021/acsomega.2c07845 (PMC10061657; doi:10.1021/acsomega.2c07845)
Supplement: Supplementary file 1 — ao2c07845_si_001.pdf [file ao2c07845_si_001.pdf]

Supporting Information

Reactive extraction of betaine from sugarbeet processing by-products

Sinem Altinisik[1,a], Hani Zeidan[1], M. Deniz Yilmaz[2], and Mustafa E. Marti[1,]*

[1] Department of Chemical Engineering, Faculty of Engineering and Natural Sciences, Konya Technical University, 42075 Konya, Türkiye
[2] Department of Basic Sciences, Faculty of Engineering, Necmettin Erbakan University, 42140 Konya, Türkiye

[a] Current address: Department of Chemical Engineering, Çanakkale Onsekiz Mart University, 17100 Çanakkale, Türkiye

[*]Corresponding author: Assoc. Prof. Dr. Mustafa Esen Marti (e-mail: memarti@ktun.edu.tr)
Current Address: Konya Technical University Phone: +90-332-223-1837 Fax: +90-332-241-0635

Table S1. Effect and significance of various parameters on the recovery of betaine from by-products of sugarbeet processing with reactive extraction.

Type of aqueous solution	Parameter	Diluent	*p*-value	*f*-value
Aqueous Betaine Solution	Contact time	1-octanol	0.0008	198.15
	Initial extractant concentration	Toluene	0.0454	8.25
		DMP	0.0249	12.26
		MIBK	0.0065	27.04
		1-octanol	0.0276	11.48
	Initial pH	Toluene	0.2278	2.29
		1-octanol	0.2188	1.83
	Temperature	Toluene	0.9818	5.00×10^{-4}
		DMP	0.8853	0.02
	Presence of sucrose	Toluene	0.0918	4.88
		DMP	0.1931	2.44
Molasses Solution	Contact time	1-octanol	0.0008	186.78
	Initial extractant concentration	Toluene	0.0043	33.98
		DMP	0.0018	54.78
		MIBK	0.0002	168.23
		1-octanol	0.0009	79.21
	Initial pH	1-octanol	0.0253	6.21
Vinasse Solution	Contact time	1-octanol	0.0160	24.35
	Initial extractant concentration	Toluene	0.0013	63.95
		DMP	0.0008	82.80
		MIBK	0.0002	159.59
		1-octanol	0.0021	50.62
	Initial pH	1-octanol	0.0328	5.22

*p-value < 0.05 and f-value > 5: significant. p-value > 0.05 and f-value < 5: insignificant.

Chemical	Structure
Betaine	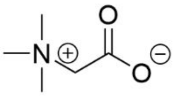
DNNDSA	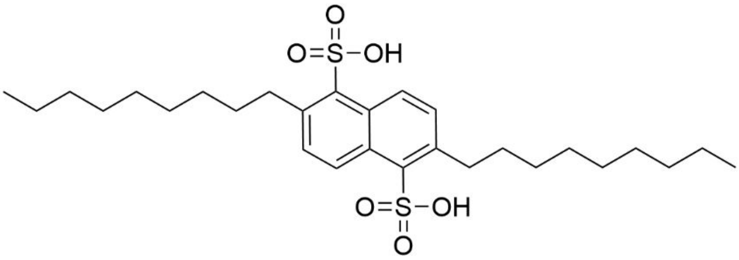
1-Octanol	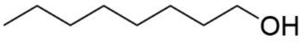
Toluene	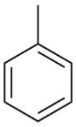
MIBK	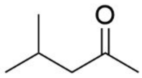
DMP	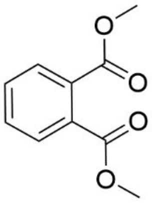

Figure S1. Structures of the chemicals used in this study

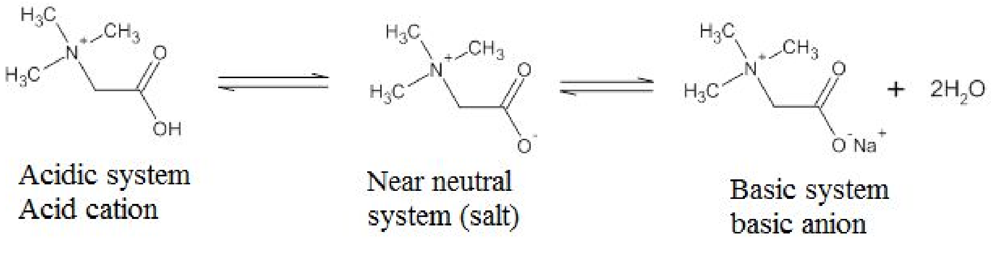

Figure S2. Behavior of betaine in acidic, neutral and basic solutions.
